# Insulin Resistance and Oxidative Stress: In Relation to Cognitive Function and Psychopathology in Drug-Naïve, First-Episode Drug-Free Schizophrenia

**DOI:** 10.3389/fpsyt.2020.537280

**Published:** 2020-11-19

**Authors:** Qi Tao, Yu Miao, Huihui Li, Xiuxia Yuan, Xufeng Huang, Yunpeng Wang, Ole A. Andreassen, Xiaoduo Fan, Yongfeng Yang, Xueqin Song

**Affiliations:** ^1^Department of Psychiatry, The First Affiliated Hospital, Zhengzhou University, Zhengzhou, China; ^2^Biological Psychiatry International Joint Laboratory of Henan/Zhengzhou University, Zhengzhou, China; ^3^Henan Psychiatric Transformation Research Key Laboratory/Zhengzhou University, Zhengzhou, China; ^4^Academy of Medical Sciences/Zhengzhou University, Zhengzhou, China; ^5^Illawarra Health and Medical Research Institute and University of Wollongong, Wollongong, NSW, Australia; ^6^Lifespan Changes in Brain and Cognition (LCBC), Department of Psychology, University of Oslo, Oslo, Norway; ^7^Norwegian Center for Mental Disorders Research (NORMENT), Institute of Clinical Medicine, University of Oslo, Oslo, Norway; ^8^Psychotic Disorders Program, UMass Memorial Medical Center, University of Massachusetts Medical School, Worcester, MA, United States; ^9^Henan Mental Hospital, The Second Affiliated Hospital of Xinxiang Medical University, Xinxiang, China; ^10^Henan Key Lab of Biological Psychiatry of Xinxiang Medical University, Xinxiang, China; ^11^International Joint Research Laboratory for Psychiatry and Neuroscience of Henan, Xinxiang, China

**Keywords:** schizophrenia, insulin resistance, oxidative stress, cognitive impairment, psychopathology

## Abstract

**Objective:** The present study aimed to examine whether insulin resistance and oxidative stress are associated with cognitive impairment in first-episode drug-free schizophrenia (SZ) patients.

**Methods:** Ninety first-episode SZ patients and 70 healthy controls were enrolled. Fasting insulin (FINS) and markers of oxidative stress [oxidized glutathione (GSSG), superoxide dismutase (SOD), nitric oxide (NO) and uric acid (UA) levels] were measured in serum before pharmacological treatment was initiated. Psychiatric symptoms and cognitive function were assessed with the Positive and Negative Syndrome Scale (PANSS) and MATRICS Consensus Cognitive Battery (MCCB), respectively. In addition, the homeostatic model assessment of insulin resistance (HOMA-IR) was also studied.

**Results:** HOMA-IR and serum levels of GSSG and NO were significantly higher in SZ patients than in healthy controls (*P* < 0.001), while the serum levels of SOD were significantly lower than in healthy controls (*P* < 0.001). HOMA-IR, GSSG and NO levels were significantly correlated to the total cognitive function scores of the patient group (*r* = −0.345,−0.369,−0.444, respectively, *P* < 0.05). But these factors were not co-related to the cognitive functions in the healthy control group. And, levels of SOD, UA were not associated with the total cognitive function scores in both the patient and the healthy control groups. NO was positively correlated with general pathological and the total score in the PANSS, and was negatively correlated with six cognitive domains (*r* = −0.316 to −0.553, *P* < 0.05).

**Conclusions:** The levels of insulin resistance and oxidative stress are elevated, and correlated with the severity of cognitive impairment in drug-naïve, first-episode SZ patients. Treatment approaches targeting on reducing insulin resistance and oxidative stress may improve cognitive function in SZ patients.

## Introduction

Schizophrenia (SZ) is a chronic severe mental illness with mainly unknown etiology, which incurs heavy burden on the persons affected, their families and the society (1). Cognitive impairment has been increasingly recognized as a core feature of SZ, and is associated with reduced social functioning, which is important for the prognosis of patients. N-methyl-D-aspartate (NMDA) receptor hypofunction has been implicated in pathophysiology of SZ (2). Previous studies have suggested that cognitive impairment is related to the hypofunction of the NMDA receptor, a type of ionic glutamate (Glu) receptor (3). Long-term potentiation (LTP) is induced through the NMDA receptor pathway to enhance learning and memory (4). When the NMDA receptor is over-activated, it causes excitatory toxicity to neural cells, leading to cell damage and death. Moreover, the proper function of the NMDA channel in the central nervous systems has been reported to be regulated by many other factors (5, 6).

Insulin resistance (IR) refers to the decreased efficiency of insulin in the promotion of glucose uptake and use. Insulin have effect on brain function, as it regulates the activity of NMDA and improves synaptic plasticity (7). When the biological efficacy of insulin decreases, the learning and memory function are reduced. Chen et al. believed that IR seems to play a role in cognitive impairment in SZ (8). Studies have found that increased IR may occur earlier in first-episode SZ patients than in healthy controls (9). Ringen (10) also proposed that IR has been associated with SZ.

Studies have found that the levels of oxidized products such as GSSG and NO are increased, and the levels of antioxidant products such as SOD are decreased in patients with schizophrenia (11), and such changes are associated with cognitive function and psychopathology (12). Studies have shown that that oxidative stress causes cognitive impairment by damaging neurons (13). The excitatory amino acid Glu and NMDA receptors are closely related to oxidative stress, which may cause cognitive impairment through the Glu-NMDAR-NO pathway (14). Glu can activate NMDA receptors (15), and produce excessive oxide and nitric oxide (NO), leading to neuronal excitotoxicity involved in the development of various neuropsychiatric diseases (16). Oxidative stress may be an intermediary mechanism of NMDA receptor dysfunction involved in the occurrence of schizophrenia. Boskovic suggested that oxidative stress may be involved in the occurrence and development of SZ (17). UA scavenges singlet oxygen and free radicals, and it can also trap peroxynitrite anions (free radicals in the ONOO –), thereby reduce the damage mediated by ONOO–. Thus, UA is an effective neuroprotective compounds (18). In addition, serum levels of UA can indicate the oxidative stress state of the body. Houlihan et al. (19) studied that UA at a high level may improve memory-related behaviors in cognitive function. However, there is no definitive conclusion on the relationship between UA and cognitive function.

Previous studies have reported an associations between IR, oxidative stress and the risk of type 2 diabetes (20, 21), but there has been little research on the associations between IR, oxidative stress and SZ. In addition, several lines of evidence showed that the antioxidant defense system may be disrupted in SZ patients, and excessive free radical production and oxidative stress damage response may be involved in the pathogenesis of SZ (22–24). Few studies have focused on the association between IR, oxidative stress and cognitive function in SZ patients.

The present study was to investigate if serum levels of biomarkers reflecting IR and oxidative stress are elevated in patients with drug-naïve, first episode SZ, and whether such biomarkers are associated with cognitive function in these patients.

## Materials and Methods

### Participants

All subjects in this study were approved by the Ethics Committee of the First Affiliated Hospital of Zhengzhou University and provided written informed consent. Inpatients 18 and 45 years old diagnosed with first-episode SZ (disease duration <2 years) were recruited. The inclusion criteria for patients were: (1) diagnosis of first episode SZ based on the Diagnostic and Statistical Manual of Mental Disorders fourth version (DSM-IV) criteria and confirmed using the Structured Clinical Interview for DSM-IV (SCID) (25); (2) never treated with antipsychotic medications or other psychotropics; (3) the PANSS total score >60 points. Exclusion criteria included: (1) diagnose neurological or other mental illnesses, autoimmune diseases, diabetes and other organic diseases; (2) alcohol or substance abuse history; (3) pregnant or lactating women; (4) have taken any antibiotics, anti-inflammatory agents or probiotics in the past month; (5) major changes in living environment or diet in the past month. Recruitment of normal-weight healthy control subjects through advertisement, they had the same exclusion criteria as the patient group; Moreover, none of them had a history of any psychiatric diseases.

### Assessments

The psychiatric symptoms of SZ were assessed in all enrolled patients using the Positive and Negative Syndrome Scale (PANSS) (26). The PANSS was administered by a professionally trained and experienced psychiatrist. All subjects received a baseline cognitive evaluation using the MATRICS Consensus Cognitive Battery (MCCB) (27). It involves seven cognitive areas: (1) Speed of Processing Information; (2) Attention and Vigilance Awareness; (3) Working Memory; (4): Verbal Learning; (5) Visual Learning; (6) Reasoning and Problem Solving; (7) Social Cognition. The MCCB scoring program generates T-scores that are standardized and corrected for age and sex (27). The “cognitive composite” is the standardized sum of the seven domains. Training, data collection and data quality assurance were implemented or supervised by experienced psychologists in accordance with the guidelines outlined in the MCCB manual (27).

### Biochemical Measurements

After all subjects were enrolled, 5 ml of venous blood was collected from the elbow under fasting condition (12 h fasting) by full-time laboratory personnel in the morning of the next day from 6:30 a.m. to 7:30 a.m. to avoid the influence of biological rhythm changes of the measured factors. Venous blood samples were collected from the elbows into EDTA anticoagulant tubes at 4°C for 10 min at 3,000 r/rain to separate the upper serum. FPG levels were measured by the glucose oxidase method and an automated analyzer (Roche Diagnostics, C8000, Germany), serum FINS levels were measured by radioimmunoassay (Elecsys 2010, Roche, Basel, Switzerland); GSSG was measured by a microenzyme method (A061-1,China); Serum SOD levels was measured using the kits and Roche automatic biochemical analyzer (Roche Diagnostics, C8000, Germany); NO levels was detected by one-step method (A013-2,China); The serum uric acid (UA)levels was measured by uricase-peroxidase method (Roche, C720, Switzerland). The current group of non-smokers had never smoked before. The homeostasis model of assessment of insulin resistance (HOMA-IR) was calculated using the following formula: HOMA-IR = FPG (mmol/L) × FINS (mu/L)/22.5 (28). Body mass index (BMI) = height/body mass^2^ (kg/m^2^).

### Statistical Analysis

SPSS 21.0 statistical software was used for all data analysis. All numeric variables data were expressed as mean ±SD. All categorical variables data were expressed as ratios and frequencies. Group comparison was performed using an unpaired *t*-test, and Chi-square test was used for testing independence among categorical variables. The correlations between IR, oxidative stress and cognitive function was computed by Pearson's correlation. two-sided *P* < 0.05 indicated that the difference was statistically significant.

## Results

Table 1 shows that there were no significant differences in age, education, gender, smoking status, and BMI between the SZ patients and healthy control groups (*P* > 0.05). In the seven domains of cognitive function, the scores of the SZ patient group were significantly lower than those of the healthy control group (*P* < 0.05, Table 1).

**Table 1:** Demographic and clinical characteristics of the study sample.

	**Patients**	**Healthy controls**	**Group comparison**	
**Characteristics**	***N* = 90 >Mean (SD)**	***N* = 70 Mean (SD)**	***t/χ2***	***p***
Age (years)	21.5 ± 7.7	23.4 ± 5.4	−1.592	0.114
Education (years)	10.4 ± 2.6	11.1 ± 2.4	−1.63	0.106
BMI (kg/m^2^)	21.5 ± 2.2	21.1 ± 2.4	1.052	0.295
Disease duration (months)	5.9 ± 6.3			
	*N* (%)	*N* (%)		
Gender			−0.397	0.692
Male	44 (48.9)	32 (45.7)		
Female	46 (51.1)	38 (54.3)		
Smoking status			1.056	0.293
Yes	5 (0.06)	7 (0.10)		
No	85 (0.94)	63 (0.90)		
PANSS-total	84.2 ± 12.7			
PANSS-positive	23.2 ± 4.8			
PANSS-negative	22.4 ± 5.9			
PANSS-general	38.5 ± 7.8			
MCCB composite score	28.6 ± 16.5	45.1 ± 19.4	−5.790	0.000
SOP	30.6 ± 9.4	51.7 ± 10.0	−12,334	0.000
CPT-IP	32.5 ± 10.9	50.7 ± 9.2	−10.201	0.000
WMS-III	38.8 ± 9.4	55.6 ± 10.6	−9.632	0.000
HVLT	37.6 ± 7.6	50.7 ± 10.0	−8.465	0.000
BVMT	39.8 ± 10.1	57.2 ± 12.1	−8.925	0.000
NAB	40.1 ± 9.8	46.1 ± 10.5	−3.351	0.001
MSCEIT	39.0 ± 11.6	56.3 ± 15.1	−7.375	0.000

*PANSS-positive, Positive Symptom; PANSS-negative, Negative Symptom; PANSS-general, General Pathological Score; PANSS-total, PANSS Total Score*.

Table 2 shows that FPG levels in SZ patients showed an increased trend but fell short of statistical significance (*t* = 1.448, *P* = 0.150); However, FINS, HOMA-IR, GSSG, and NO levels were higher in the SZ patients than in the healthy controls (*P* < 0.001). On the other hand, the serum SOD levels were lower in the SZ patients than in the healthy controls (*t* = −3.703, *P* < 0.001).

**Table 2:** Comparison of insulin resistance and oxidative stress measures between the two groups.

**Items**	**Patient group (*N* = 90)**	**Healthy control group (*N* = 70)**	***t***	***P***
**BIOCHEMICAL MEASURES**				
FPG (mmol/L)	4.4 ± 0.5	4.3 ± 0.7	1.448	0.150
FINS (mmol/L)	11.1 ± 3.3	6.1 ± 1.1	10.338	0.000
HOMA-IR (mU/L)	2.2 ± 0.7	1.2 ± 0.3	10.790	0.000
GSSG	30.9 ± 7.6	16.8 ± 5.8	11.667	0.000
NO	103.9 ± 54.5	32.2 ± 33.6	8.839	0.000
SOD	198.6 ± 43.1	231.7 ± 29.1	−3.703	0.000
UA	292.2 ± 87.5	273.6 ± 60.1	1.303	0.195

*FPG, fasting plasma glucose; FINS, fasting insulin; HOMA-IR, insulin resistance index; GSSG, oxidized glutathione; NO, nitric oxide; SOD, superoxide dismutase; UA, uric acid*.

Table 3 shows that HOMA-IR, GSSG and NO were significantly correlated to the total cognitive function scores of the patient group (*r* = −0.345, −0.369, −0.444, respectively, *P* < 0.05, Table 3). In a multiple regressing model including HOMA-IR, GSSG, NO as predictors, and the total MCCB score as the dependent variable, we found that only HOMA-IR had an effect on the MCCB composite score (*t* = −2.321, *P* < 0.05).

**Table 3:** Insulin resistance, oxidative stress, and cognitive function.

**Indicators**	**HOMA-IR**		**GSSG**		**NO**		**SOD**		**UA**	
	***r***	***P***	***r***	***P***	***r***	***P***	***r***	***P***	***r***	***P***
MCCB composite score (Patient group)	−0.345	0.001	−0.369	0.003	−0.444	0.000	0.200	0.143	−0.113	0.350
MCCB composite score (Healthy control group)	0.020	0.891	−0.153	0.230	0.001	0.997	0.002	0.992	0.118	0.412

Table 4 shows that within the patient groups, the NO levels were positively correlated with the general pathological score and total score in the PANSS assessment (*r* = 0.323,0.375, respectively, *P* < 0.05, Table 4). After controlling for age, gender, disease duration and smoking status, we found that these biological indicators (FINS, HOMA-IR, GSSG, NO, SOD, UA) showed no correlation with the scores of PANSS (positive symptom score, negative symptom score, general pathology score, and PANSS total score) (*P* > 0.001).

**Table 4:** Correlation of FINS, HOMA-IR, GSSG, NO, SOD, and UA values with PANSS in patient group (*N* = 90).

**Indicators**	**FINS**		**HOMA-IR**		**GSSG**		**NO**		**SOD**		**UA**	
	***r***	***P***	***r***	***P***	***r***	***P***	***r***	***P***	***r***	***P***	***r***	***P***
PANSS-positive	0.041	0.763	0.146	0.283	0.119	0.401	0.159	0.260	−0.081	0.591	−0.191	0.176
PANSS-negative	0.012	0.930	0.020	0.886	−0.082	0.562	0.213	0.130	−0.125	0.409	0.012	0.931
PANSS-general	0.078	0.568	0.013	0.924	0.011	0.936	0.323	0.020	0.024	0.874	−0.142	0.317
PANSS-total	0.065	0.636	0.059	0.663	0.016	0.908	0.375	0.006	−0.083	0.585	−0.138	0.331

## Discussion

Previous studies have reported associations between IR, oxidative stress and the risk of type 2 diabetes (20), but there has been little research on the associations between IR, oxidative stress and SZ. In addition, few studies have focused on the association between IR, oxidative stress and cognitive function in SZ patients. Our study found that serum marker levels for insulin resistance and oxidative stress were increased compared to healthy controls in first-episode untreated SZ patients, and that the levels were associated with cognitive function impairment. We also found correlations between several indicators of oxidative stress and clinical symptoms in the SZ patient group. To our knowledge, the present study is the first to show associations between cognitive performance and markers of IR and oxidative stress in first-episode untreated SZ patients.

Serum FINS and HOMA-IR were increased in first-episode SZ patients, consistent with the previous study by Spelman (9). Other published studies have shown that the development of IR may be caused by decreased insulin tyrosine kinase receptor activity, abnormal insulin signaling, decreased glucose transportation, decreased glucose phosphorylation and decreased glycogen synthase activity (29). As a result of IR, the activity of the cholinergic system in the hippocampus and other brain regions is significantly decreased, which can lead to neuronal degeneration and aggravated cognitive impairment. Our results showed that FINS levels and HOMA-IR were positively correlated with the severity of cognitive impairment in SZ patients. This effect may be related to the following aspects: (1) IR may be accompanied by insulin-like growth factor-1 (IGF-1) resistance, which competitively inhibits the binding of insulin to the insulin receptor (30). Other studies have reported elevated plasma FINS levels and decreased IGF-1 in SZ patients (31). (2) High FINS levels can disturb the insulin signaling pathway and decrease metalloproteinase (IDE) levels, leading to intracellular and extracellular Amyloid- β deposition, and causing neuronal degeneration. (3) IR can promote neuronal inflammation by enhancing the release of pro-inflammatory cytokines such as IL-1α, IL-1β, IL-6, and TNF-α. (4) Long-term IR can increase the content of highly active oxidative groups and decrease the activity of antioxidant enzymes, causing neuronal cell apoptosis (32).

In the present study, we found that the levels of oxidized products (GSSG, and NO) were significantly increased in SZ patients, while SOD levels were significantly decreased, and that these levels were correlated to some cognitive function domains. Numerous studies have revealed an imbalance between the oxidative and antioxidant systems in SZ patients (33). Evidenced by increased GSSG and NO levels accompanied by decreased SOD levels (34), and such an imbalance may correlate with impaired cognitive functions (12). NO, as a signaling molecule, may be involved in the impairment of cognitive function in SZ patients through the following mechanisms: (1) activation of the hypothalamus- pituitary-adrenal axis, secretion of prolactin, and corticosteroids, and elevation of hormones levels and thus activating the dopaminergic neurons in the hypothalamus, causing positive symptoms and cognitive impairment (35); (2) NMDA receptor activation, which is involved in the release of Glu and dopamine, and causes cognitive deficits and mental disorders (36); and (3) reduction in nitrite ions and superoxide anions, which react to the formation of peroxynitrite anions, leading to neurotoxic effects.

Previous studies have suggested that the antioxidant defense system is disrupted in SZ patients, and excessive free radical production and oxidative stress damage response may be involved in the pathogenesis of SZ (22, 23). SOD and UA are both effective indicators of antioxidant capacity, and SOD as an enzyme can effectively scavenge oxygen free radicals in the body, representing an important component of the free radical defense system. Our results showed that SOD levels were significantly decreased in SZ patients compared with healthy control subjects, which was consistent with the study by Reyazuddin (37), indicating that there may be a high level of oxidative stress in SZ patients. Other studies noted that the presence of the oxidative stress indicators SOD and NO in first-episode SZ patients is associated with cognitive impairment, consistent with the results of the present study. Our study did not find a correlation between UA levels and cognitive function impairment in SZ patients. whether SZ patients have a lower level of UA remains controversial (38–40).

Hyperglycemia, which mediates oxidative stress by directly or indirectly activating the diacylglycerol (DAG) -PKC pathway, is the main source of oxidative stress (41). Studies have shown that the PKC-D subtype is a potential activator of nicotinamide adenine dinucleotide phosphate (NADPH) oxidase, and the activation of NADPH oxidase can increase the production of reactive oxygen species(ROS) (42). Normally, insulin receptor substrates (IRS-1 and IRS-2) are mainly distributed in low density microsomes (LDMs). However, but upon activating these receptors, phosphatidylinositol 3-kinase (P13K) is recruited to LDMs (43). Oxidative stress can interfere with P13K migration from the cytoplasm to LDMs, thereby inducing IR. Thus, oxidative stress reduces the recruitment of P13K by interfering with the phosphorylation of insulin receptor substrates, thereby inducing IR. On the other hand, High levels of free fatty acids lead to an increase in ROS and reactive nitrogen species (RNS), thereby activating the oxidative stress mechanisms that damage DNA, proteins and lipids. Additionally, high levels of free fatty acids can activate a range of intracellular signaling pathways that are closely related to IR and other cellular functions. The dysregulation of free fatty acids leads to neurodegenerative disease by promoting the phosphorylation of tau in the hippocampus (44). Insulin inhibits the activity of glycogen synthesis kinase-3β (GSK-3β), which phosphorylates tau, pyruvate dehydrogenase, and glycogen synthase. GSK-3β activation under insulin deficiency or IR promotes the phosphorylation of tau and the inactivation of pyruvate dehydrogenase and glycogen, affecting energy metabolism and acetylcholine synthesis. Thus, the interplay between IR and oxidative stress factors can synergistically impair cognitive functions in the SZ patients (45).

This study has several advantages. Our results were based on a relatively large number of drug-naïve SZ patients. The status of being drug-naïve and first episodes are important in removing the impact of medications on the associations between IR and oxidative stress and cognitive impairment in SZ patients. However, the present study also has limitations. Our results were based on analysis of cross-sectional samples. Future follow-up studies that have measurements at multiple time points may strengthen our conclusions. In addition, unmeasured confounders in the present study may have some impact on our results thus future replication studies are warranted.

In summary, in the present work, we report abnormal IR and oxidative stress factors in first-episode untreated SZ patients, and show how they are correlated to on cognitive function. There was an association between IR and oxidative stress in the patients. The current findings need to be replicate, but suggest that IR may be a peripheral biological marker of cognitive dysfunction development in SZ patients, and could play a role in the pathological disease processes.

## Data Availability Statement

The datasets generated for this study will not be made publicly available this data is confidential.

## Ethics Statement

The studies involving human participants were reviewed and approved by Ethics Committee of the First Affiliated Hospital of Zhengzhou University. The patients/participants provided their written informed consent to participate in this study. Written informed consent was obtained from the individual(s) for the publication of any potentially identifiable images or data included in this article.

## Author Contributions

XS and YY contributed the conception and the design of the study. YM and HL were in charge of conducting clinical assessment and collecting fasting blood samples. QT, YM, and XY undertook the statistical analysis. QT, YM, and YY wrote the draft of the paper. YM revised the paper. XH, YW, OA, and XF were responsible for supervision and reviewing. All authors approved the submitted version.

## Conflict of Interest

The authors declare that the research was conducted in the absence of any commercial or financial relationships that could be construed as a potential conflict of interest.

## References

[1] Owen MJ Sawa A Mortensen PB. Schizophrenia. Lancet. (2016) 388:86–97. 10.1016/S0140-6736(15)01121-6 26777917 PMC4940219

[2] Nakazawa K Sapkota K The origin of NMDA receptor hypofunction in schizophrenia. Pharmacol Ther. (2019) 2019:107426 10.1016/j.pharmthera.2019.107426 PMC6981256 31629007

[3] Li N Liu RJ Dwyer JM Banasr M Lee B Son H. Glutamate N-methyl-D-aspartate receptor antagonists rapidly reverse behavioral and synaptic deficits caused by chronic stress exposure. Biol Psychiatry. (2011) 69:754–61. 10.1016/j.biopsych.2010.12.015 21292242 PMC3068225

[4] Kerchner GA Nicoll RA. Silent synapses and the emergence of a postsynaptic mechanism for LTP. Nat Rev Neurosci. (2008) 9:813–25. 10.1038/nrn2501 18854855 PMC2819160

[5] Burnouf S Martire A Derisbourg M Laurent C Belarbi K Leboucher A. NMDA receptor dysfunction contributes to impaired brain-derived neurotrophic factor-induced facilitation of hippocampal synaptic transmission in a Tau transgenic model. Aging Cell. (2013) 12:11–23. 10.1111/acel.12018 23082852

[6] Wu RQ Lin CG Zhang W Lin XD Chen XS Chen C. Effects of risperidone and paliperidone on brain-derived neurotrophic factor and N400 in first-episode schizophrenia. Chinese Med J-Peking. (2018) 131:2297–301. 10.4103/0366-6999.241802 30246715 PMC6166445

[7] Filippi BM Abraham MA Yue JT Lam TK. Insulin and glucagon signaling in the central nervous system. Rev Endocr Metab Disord. (2013) 14:365–75. 10.1007/s11154-013-9258-4 23959343

[8] Zhao WQ Chen H Quon MJ Alkon DL. Insulin and the insulin receptor in experimental models of learning and memory. Eur J Pharmacol. (2004) 490:71–81. 10.1016/j.ejphar.2004.02.045 15094074

[9] Spelman LM Walsh PI Sharifi N Collins P Thakore JH. Impaired glucose tolerance in first-episode drug-naive patients with schizophrenia. Diabetic Med. (2007) 24:481–5. 10.1111/j.1464-5491.2007.02092.x 17381506

[10] Ringen PA Engh JA Birkenaes AB Dieset I Andreassen OA. Increased mortality in schizophrenia due to cardiovascular disease - a non-systematic review of epidemiology, possible causes, and interventions. Front Psychiatr. (2014) 5:137. 10.3389/fpsyt.2014.00137 25309466 PMC4175996

[11] Bitanihirwe BK Woo TU. Oxidative stress in schizophrenia: an integrated approach. Neurosci Biobehav Rev. (2011) 35:878–93. 10.1016/j.neubiorev.2010.10.008 20974172 PMC3021756

[12] Pitsikas N. The role of nitric oxide donors in schizophrenia: Basic studies and clinical applications. Eur J Pharmacol. (2015) 766:106–13. 10.1016/j.ejphar.2015.09.045 26435027

[13] Huang TT Leu D Zou Y. Oxidative stress and redox regulation on hippocampal-dependent cognitive functions. Arch Biochem Biophys. (2015) 576:2–7. 10.1016/j.abb.2015.03.014 25797440 PMC4456256

[14] Hardingham GE Do KQ. Linking early-life NMDAR hypofunction and oxidative stress in schizophrenia pathogenesis. Nat Rev Neurosci. (2016) 17:125–34. 10.1038/nrn.2015.19 26763624

[15] Parpura V Basarsky TA Liu F Jeftinija K Jeftinija S Haydon PG. Glutamate-mediated astrocyte-neuron signalling. Nature. (1994) 369:744–7. 10.1038/369744a0 7911978

[16] Shim S Shuman M Duncan E. An emerging role of cGMP in the treatment of schizophrenia: a review. Schizophr Res. (2016) 170:226–31. 10.1016/j.schres.2015.11.015 26706197

[17] Boskovic M Vovk T Kores Plesnicar B Grabnar I. Oxidative stress in schizophrenia. Curr Neuropharmacol. (2011) 9:301–12. 10.2174/157015911795596595 22131939 PMC3131721

[18] Squadrito GL Cueto R Splenser AE Valavanidis A Zhang H Uppu RM. Reaction of uric acid with peroxynitrite and implications for the mechanism of neuroprotection by uric acid. Arch Biochem Biophys. (2000) 376:333–7. 10.1006/abbi.2000.1721 10775420

[19] Houlihan LM Wyatt ND Harris SE Hayward C Gow AJ Marioni RE. Variation in the uric acid transporter gene (SLC2A9) and memory performance. Hum Mol Genet. (2010) 19:2321–30. 10.1093/hmg/ddq097 20197412

[20] Hirsch GE Heck TG. Inflammation, oxidative stress and altered heat shock response in type 2 diabetes: the basis for new pharmacological and non-pharmacological interventions. Arch Physiol Biochem. (2019) 2019:1–15. 10.1080/13813455.2019.1687522 31746233

[21] Dimova R Chakarova N Grozeva G Kirilov G Tankova T. The relationship between glucose variability and insulin sensitivity and oxidative stress in subjects with prediabetes. Diabetes Res Clin Pract. (2019) 158:107911. 10.1016/j.diabres.2019.107911 31707004

[22] Zhang XY Chen DC Xiu MH Tang W Zhang F Liu L. Plasma total antioxidant status and cognitive impairments in schizophrenia. Schizophr Res. (2012) 139:66–72. 10.1016/j.schres.2012.04.009 22555016

[23] Matsuzawa D Hashimoto K Hashimoto T Shimizu E Watanabe H Fujita Y. Association study between the genetic polymorphisms of glutathione-related enzymes and schizophrenia in a japanese population. Am J Med Genet B. (2009) 150:86–94. 10.1002/ajmg.b.30776 18449862

[24] Solberg DK Refsum H Andreassen OA Bentsen H. A five-year follow-up study of antioxidants, oxidative stress and polyunsaturated fatty acids in schizophrenia. Acta Neuropsychiatr. (2019) 31:202–12. 10.1017/neu.2019.14 31178002

[25] Runeson BS Rich CL. Diagnostic and statistical manual of mental disorders, 3rd ed. (DSM-III), adaptive functioning in young Swedish suicides. Ann Clin Psychiatry. (1994) 6:181–3. 10.3109/10401239409149001 7881498

[26] Kay SR Fiszbein A Opler LA. The positive and negative syndrome scale (PANSS) for schizophrenia. Schizophr Bull. (1987) 13:261–76. 10.1093/schbul/13.2.261 3616518

[27] Nuechterlein KH Green MF Kern RS Baade LE Barch DM Cohen JD. The MATRICS consensus cognitive battery, part 1: test selection, reliability, and validity. Am J Psychiatry. (2008) 165:203–13. 10.1176/appi.ajp.2007.07010042 18172019

[28] Hermans MP Levy JC Morris RJ Turner RC. Comparison of insulin sensitivity tests across a range of glucose tolerance from normal to diabetes. Diabetologia. (1999) 42:678–87. 10.1007/s001250051215 10382587

[29] Djiogue S Nwabo Kamdje AH Vecchio L Kipanyula MJ Farahna M Aldebasi Y. Insulin resistance and cancer: the role of insulin and IGFs. Endocr Relat Cancer. (2013) 20:R1–17. 10.1530/ERC-12-0324 23207292

[30] Friedrich N Jorgensen T Juul A Spielhagen C Nauck M Wallaschofski H. Insulin-like growth factor I and anthropometric parameters in a Danish population. Exp Clin Endocrinol Diabetes. (2012) 120:171–4. 10.1055/s-0031-1301289 22402920

[31] Venkatasubramanian G Chittiprol S Neelakantachar N Naveen MN Thirthall J Gangadhar BN Insulin and insulin-like growth factor-1 abnormalities in anti psychotic-naive schizophrenia. Am J Psychiat. (2007) 164:1557–60. 10.1176/appi.ajp.2007.07020233 17898347

[32] Mota M Banini BA Cazanave SC Sanyal AJ. Molecular mechanisms of lipotoxicity and glucotoxicity in nonalcoholic fatty liver disease. Metabolism. (2016) 65:1049–61. 10.1016/j.metabol.2016.02.014 26997538 PMC4931958

[33] Gu F Chauhan V Chauhan A. Glutathione redox imbalance in brain disorders. Curr Opin Clin Nutr Metab Care. (2015) 18:89–95. 10.1097/MCO.0000000000000134 25405315

[34] Ballesteros A Jiang P Summerfelt A Du XM Chiappelli J O'Donnell P. No evidence of exogenous origin for the abnormal glutathione redox state in schizophrenia. Schizophr Res. (2013) 146:184–9. 10.1016/j.schres.2013.02.001 23466187 PMC3622807

[35] Bruehl H Rueger M Dziobek I Sweat V Tirsi A Javier E. Hypothalamic-pituitary-adrenal axis dysregulation and memory impairments in type 2 diabetes. J Clin Endocr Metab. (2007) 92:2439–45. 10.1210/jc.2006-2540 17426095

[36] Maia-de-Oliveira JP Kandratavicius L Nunes EA Machado-de-Sousa JP Hallak JE Dursun SM. Nitric oxide's involvement in the spectrum of psychotic disorders. Curr Med Chem. (2016) 23:2680–91. 10.2174/0929867323666160721144549 27450675

[37] Reyazuddin M Azmi SA Islam N Rizvi A. Oxidative stress and level of antioxidant enzymes in drug-naive schizophrenics. Indian J Psychiat. (2014) 56:344–9. 10.4103/0019-5545.146516 25568474 PMC4279291

[38] Pae CU Paik IH Lee C Lee SJ Kim JJ Lee CU. Decreased plasma antioxidants in schizophrenia. Neuropsychobiology. (2004) 50:54–6. 10.1159/000077942 15179021

[39] Yao JK Dougherty GG Jr Reddy RD Keshavan MS Montrose DM Matson WR. Homeostatic imbalance of purine catabolism in first-episode neuroleptic-naive patients with schizophrenia. PLoS ONE. (2010) 5:e9508. 10.1371/journal.pone.0009508 20209081 PMC2831068

[40] Reddy R Keshavan M Yao JK. Reduced plasma antioxidants in first-episode patients with schizophrenia. Schizophr Res. (2003) 62:205–12. 10.1016/S0920-9964(02)00407-3 12837516

[41] Ahmad FK He ZH King GL. Molecular targets of diabetic cardiovascular complications. Curr Drug Targets. (2005) 6:487–94. 10.2174/1389450054021990 16026267

[42] Ewald CY. Redox Signaling of NADPH oxidases regulates oxidative stress responses, immunity and aging. Antioxidants. (2018) 7:10. 10.3390/antiox7100130 30274229 PMC6210377

[43] Kriauciunas KM Myers MG Jr Kahn CR. Cellular compartmentalization in insulin action: altered signaling by a lipid-modified IRS-1. Mol Cell Biol. (2000) 20:6849–59. 10.1128/MCB.20.18.6849-6859.2000 10958681 PMC86221

[44] Schubert M Brazil DP Burks DJ Kushner JA Ye J Flint CL. Insulin receptor substrate-2 deficiency impairs brain growth and promotes tau phosphorylation. J Neurosci. (2003) 23:7084–92. 10.1523/JNEUROSCI.23-18-07084.2003 12904469 PMC6740672

[45] Gasparini L Netzer WJ Greengard P Xu HX. Does insulin dysfunction play a role in Alzheimer's disease? Trends Pharmacol Sci. (2002) 23:288–93. 10.1016/S0165-6147(02)02037-0 12084635

